# Genomewide identification of a novel six‐LncRNA signature to improve prognosis prediction in resectable hepatocellular carcinoma

**DOI:** 10.1002/cam4.1854

**Published:** 2018-10-30

**Authors:** Ying Wu, Peng‐Shuo Wang, Ben‐Gang Wang, Lu Xu, Wan‐Xia Fang, Xiao‐Fang Che, Xiu‐Juan Qu, Yun‐Peng Liu, Zhi Li

**Affiliations:** ^1^ Department of General Practice The First Hospital, China Medical University Shenyang China; ^2^ Department of Psychology The First Hospital, China Medical University Shenyang China; ^3^ Department of Hepatobiliary Surgery The First Hospital, China Medical University Shenyang China; ^4^ Department of Medical Oncology The First Hospital, China Medical University Shenyang China; ^5^ Key Laboratory of Anticancer Drugs and Biotherapy of Liaoning Province the First Hospital of China Medical University Shenyang China

**Keywords:** biomarker, hepatocellular carcinoma, LncRNA, prognosis

## Abstract

The current prognostic long noncoding RNA (lncRNA) signatures for hepatocellular carcinoma (HCC) are still controversial and need to be optimized by systematic bioinformatics analyses with suitable methods and appropriate patients. Therefore, we performed the study to establish a credible lncRNA signature for HCC outcome prediction and explore the related mechanisms. Based on the lncRNA profile and the clinical data of carefully selected HCC patients (n = 164) in TCGA, six of 12727 lncRNAs, MIR22HG, CTC‐297N7.9, CTD‐2139B15.2, RP11‐589N15.2, RP11‐343N15.5, and RP11‐479G22.8 were identified as the independent predictors of patients’ overall survival in HCC by sequential univariate Cox and 1000 times Cox LASSO regression with 10‐fold CV, and multivariate Cox analysis with 1000 times bootstrapping. In the Kaplan‐Meier analysis with patients trichotomized by the six‐lncRNA signature, high‐risk patients showed significantly shorter survival than mid‐ and low‐risk patients (log‐rank test *P* < 0.0001). According to the ROCs, the six‐lncRNA signature showed superior predictive capacity than the two existing four‐lncRNA combinations and the traditional prognostic clinicopathological parameter TNM stage. Furthermore, low MIR22HG and CTC‐297N7.9, but high CTD‐2139B15.2, RP11‐589N15.2, RP11‐343N15.5, and RP11‐479G22.8, were, respectively, demonstrated to be related with the malignant phenotypes of HCC. Functionally, the six lncRNAs were disclosed to involve in the regulation of multiple cell cycle and stress response‐related pathways via mediating transcription regulation and chromatin modification. In conclusion, our study identified a novel six‐lncRNA signature for resectable HCC prognosis prediction and indicated the underlying mechanisms of HCC progression and the potential functions of the six lncRNAs awaiting further elucidation.

## 1 INTRODUCTION

Hepatocellular carcinoma (HCC), accounting for 70% to 90% of primary liver cancer, is the fifth most common malignancy and the second leading cause of cancer death worldwide.^1^, ^2^, ^3^ Although great progress has been made on the diagnosis, treatment, and prognosis dictation of HCC, the clinical outcome is still unsatisfied.^3^, ^4^ The declaration of prognostic biomarkers might be helpful for the optimization of treatment and thus the improvement of patients' prognosis.^2^ Nonetheless, HCC, unlike for other tumor types, is a special disease with two competing death causes including cirrhosis and cancer, the combination of which decides the variations of HCC biological progression and makes the prognosis prediction highly complex.^2^, ^5^, ^6^, ^7^, ^8^ The identification of prognostic biomarkers should be integrated with the analysis of cancer stage, and the general status of both host patients and underlying liver.^2^, ^5^, ^7^ Besides tumor extents such as tumor size, grade, and stage, it is believed that liver fibrosis and function level, patients’ performance status, and postsurgical residual status are essential, and AFP and hepatitis activities are additional indicators for HCC prognosis.^2^, ^5^, ^7^, ^8^, ^9^

The development of high‐throughput sequencing techniques and bioinformatics methods has disclosed the potential prognostic value of genomic biomarkers including long noncoding RNAs (lncRNAs).^10^ LncRNAs are a class of noncoding RNAs with lengths longer than 200 nucleotides, show no capacity of protein coding, but might regulate gene expression at genetic and epigenetic levels.^11^ So far, variety of lncRNAs or lncRNA groups have been uncovered might dictate HCC prognosis by multiple studies,^10^, ^12^, ^13^, ^14^, ^15^, ^16^, ^17^ most of which are about the functional roles and working mechanisms of single lncRNAs,^14^, ^15^, ^16^ and totally two (by Wang et al in 2017 and Ma et al in 2018) are found to describe the prognostic significance of multiple‐lncRNA signature in HCC.^13^, ^17^ However, we should be wary of the conclusions of the previous studies for the following reasons.

First, most previous studies screened out survival related lncRNAs based on the differential profile between cancer and noncancerous samples.^12^, ^13^, ^14^, ^15^, ^16^ The method could miss considerable survival information in The Cancer Genome Atlas (TCGA) datasets including some important prognostic genes,^18^ and lncRNAs as well. Second, most researchers did not perform preanalysis case selection,^13^, ^16^, ^17^ whereas using an uncurated TCGA dataset without attention to sample characteristics can lead to false associations and undermine the application of the conclusions.^19^ The patients with the diagnosis of non‐HCC liver cancer (cholangiocarcinoma), pathological metastasis, and postsurgical residual carcinoma, the history of neoadjuvant treatment, and too short survival time are considered to have distinct biological procedures and progression mechanisms, and should be excluded from the prognostic analysis.^8^, ^19^, ^20^ Third, most studies performed the analysis by mixing resectable and unresectable cancers.^13^, ^14^, ^15^, ^16^, ^17^ While resectable and unresectable HCCs are demonstrated to have entirely distinct prognosis and recommended with completely different treatment strategies and staging systems (the Union for International Cancer Control (UICC)/the American Joint Cancer Committee (AJCC) vs the Cancer of the Liver Italian Program (CLIP)/the Barcelona Clinic Liver Cancer (BCLC)),^2^, ^3^, ^5^, ^6^, ^7^, ^8^, ^21^ thus should be analyzed distinctively for the development of prognostic models. Fourth, most published prognostic lncRNAs and the two established four‐lncRNA signatures were identified without evaluating the relationship with other known promising HCC prognostic factors, such as the fibrosis and function levels of liver, and the performance status and serum parameters of patients,^13^, ^14^, ^15^, ^16^, ^17^ all of which are considered vital features of HCC.^2^ Fifth, the candidate prognosis‐related lncRNAs in most previous studies were screened out by uni/multivariate Cox analysis,^12^, ^13^, ^14^, ^15^, ^16^, ^17^ the accuracy of which is considered inferior to penalized Cox regression for small‐sample and high‐dimensional data.^22^

In the current study based on appropriate selection of patients and comprehensive analysis of genetic profile and clinicopathological parameters (CPPs), we develop a novel six‐lncRNA signature that could well predict patients’ prognosis in resectable HCC by combining integrated bioinformatics tools with multiple gene‐profiling datasets. According to the receiver operating characteristics (ROCs), the six‐lncRNA signature shows better prediction accuracy than the previously discovered lncRNA groups and the traditional prognostic tumor‐node‐metastasis (TNM) stage. Subsequent gene set enrichment analysis (GSEA), gene‐set‐lncRNA network construction, and Gene Ontology‐molecular function (GO‐MF) assays for lncRNA‐related mRNAs disclose that these lncRNAs are involved in the vital processes associated with HCC progression. The results may enrich our knowledge on the progression mechanisms of HCC and also present a six‐lncRNA signature as the potential prognosis biomarker for resectable HCC.

## 2 MATERIALS AND METHODS

### 2.1 Data source and processing

The expression values of lncRNA based on the Reads Per Kilobases per Million mapped reads (RPKM) were downloaded from the Atlas of Noncoding RNAs in Cancer (TANRIC) in TCGA Liver Hepatocellular Carcinoma (LIHC) database. The mRNA expression profile, as well as the phenotypes and prognostic data, was downloaded from the University of California Santa Cruz (UCSC) xena website (https://xena.ucsc.edu/).

All of the included cases have primary HCC, definite report of survival status, and lncRNAs’ expression values, whereas those with the diagnosis of cholangiocarcinoma, pathological metastasis, and postsurgical residual carcinoma (R1 and R2), the survival duration not longer than 30 days, and the history of neoadjuvant treatment were excluded for further analysis.

LncRNAs were selected based on the following criteria according to the expression values and the calculated median and standard deviation (SD). First, lncRNAs with nonzero values in more than 66.7% of the cases were included. Second, the median and SD of the lncRNA should be larger than 1. Third, the lncRNAs were ranked by SDs and those with the SD larger than the median of SDs were included.

### 2.2 Cox survival analysis and least absolute shrinkage and selection operator (LASSO) regression with 10‐fold cross‐validation

The prognostic value of each lncRNA was firstly calculated in the univariate Cox analysis using R/survival package, and the lncRNAs with *P* < 0.01 were selected as seed lncRNAs for Cox LASSO regression with 10‐fold cross‐validation (CV).

LASSO is the penalized regression that uses an L1 penalty to shrink regression coefficients toward zero, thereby eliminate a number of variables based on the principle that the larger the penalty the fewer predictors selected. Thus, the seed lncRNAs with nonzero coefficients were considered as potential prognostic predictors. By 1000 iterations of Cox LASSO regression with 10‐fold CV using the R package glmnet (with the default parameter “standardize = T”),^23^ the seed lncRNAs were shrunk into multiple‐lncRNA sets. The lncRNA sets including lncRNAs with nonzero coefficients were potential prognostic models, whereas those with no lncRNA showing nonzero coefficients were designated as “not available (NA)” for prognostic prediction. The potential prognostic lncRNAs in the most common prognostic lncRNA set and with at last 600 occurrences were applied into further analysis.

To identify the prognostic value of the lncRNAs, multi‐variate Cox regression with 1000 times bootstrapping was further performed using R/survival package based on each “significant” lncRNA disclosed in the above steps, and the AJCC TNM stage was used as the adjustment factor. A lncRNA with *P* < 0.05 was defined as significant. The corresponding hazard ratio (HR), 95% confidence interval (CI), and *P* value were collected.

### 2.3 Kaplan‐Meier (K‐M) and ROC(*t*) curves with the novel‐identified lncRNA signature

Based on the afore‐established multivariate Cox model, the prognostic‐lncRNA linear predictor of each patient was calculated based on the value of coefficient and the expression level of the corresponding covariate: linear predictor = 0.79 × EXPRESSION_MIR22HG_ + 1.05 × EXPRESSION_CTD‐2139B15.2_ + 0.73 × EXPRESSION_CTC‐297 N7.9_ + 1.16 × EXPRESSION_RP11‐589 N15.2_ + 1.10 × EXPRESSION_RP11‐343N15.5_ + 1.12 × EXPRESSION_RP11‐479G22.8_. To confirm the prognostic significance of the candidate lncRNA signature, patients were trichotomized into three groups based on the prognostic‐lncRNA linear predictor (cutoff values: 33.33 and 66.67 quantile), and then, the K‐M curve was plotted, and the log‐rank test was calculated.

Furthermore, we calculated the resulting area under the curve (AUC) every year based on the time‐dependent ROC curves using previously reported method^24^, ^25^ and plotted the AUC (*t*) curves to compare the prediction accuracy of our candidate lncRNA prediction model with the other two published multiple‐lncRNA combinations and the traditional CPPs. The traditional CPPs with missing values above 25% were excluded, while the CPPs with enough available data (age, sex, grade, and TNM stage) were firstly evaluated for the prognostic significance using K‐M curve and log‐rank test, and the statistically significant one (TNM stage) was applied for further study.

### 2.4 Correlation analysis between each lncRNA and CPPs

The associations of each lncRNA with the CPPs were further analyzed in patients with available data. For the categorical variables, such as sex, Eastern Cooperative Oncology Group Performance Status Score (ECOG PS), risk factor, Child‐Pugh grade, ISHAK score, tumor grade, T classification, and TNM stage, Spearman correlation analysis, Wilcoxon sum rank test or Kruskal‐Wallis test was used as appropriate. For the continuous variables including age, body mass index (BMI), tumor weight, and the serum level of alpha‐fetoprotein (AFP), albumin (ALB), creatinine, platelet (PLT), and prothrombin time (PT), Pearson correlation analysis was used. Furthermore, Benjamini‐Hochberg procedure was used to control the false discovery rate (FDR). *P* < 0.05 and FDR <0.3 was defined as statistically significant. Because of the dimension inconsistency in source data of ALB and creatinine, we used adjusted values for the further analysis, and the adjusted values were calculated as follows: ALB_average_ (or creatinine_average_) = (upper limit of normal value + lower limit of normal value)/2, ALB_adjust_ = (ALB_measure_−ALB_average_)/ALB_average_, creatinine_adjust_ = (creatinine_measure_−creatinine_average_)/creatinine_average_.

### 2.5 GSEA and gene‐set‐lncRNA network construction with each of the lncRNAs

Based on the mRNA profile of the 20 530 genes downloaded from UCSC database, GSEA was performed by the JAVA program (https://www.broadinstitute.org/gsea) to identify the lncRNA‐related gene sets using MSigDB H: hallmark gene sets as functional gene sets and the expression level of each lncRNA as phenotype. After performing 1000 permutations, the first 20 gene sets with FDR *q* < 0.25 and *P* < 0.05 were considered to be significantly enriched. The gene‐set‐lncRNA network and the corresponding heat map were then constructed with R/igraph and R/heat map package.

### 2.6 lncRNA‐related mRNA extraction and GO‐MF analysis

To predict the molecular function of each candidate lncRNA, lncRNA‐related mRNAs were firstly filtered out by Pearson correlation analysis with TCGA dataset (*P* ‐ and FDR *q*‐value < 0.001) and then applied into further GO‐MF analysis using the Bioconductor “clusterProfiler” package with the statistical significance standard of *P*‐ and FDR *q*‐value <  0.05.

## 3 RESULTS

### 3.1 Basic characteristics of patients

**Figure 1:**
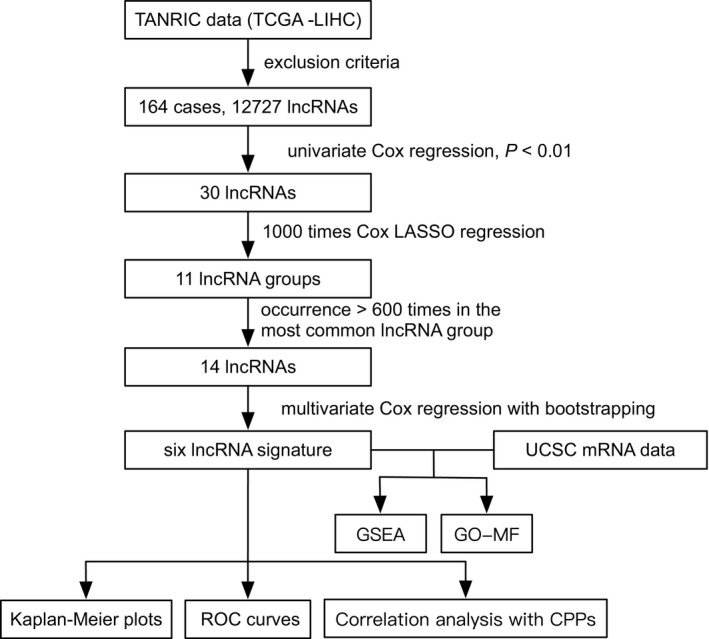
Analysis of flowchart illustrates the exploration procedure for the HCC prognostic lncRNAs and the related mechanisms

The analysis procedure of the current study is shown in Figure 1. The basic characteristics of the patients are listed in Table S1. In TCGA dataset, there were totally 164 HCC patients with the available data (Table S2) for the further analysis. Most patients were male (65.9%), in relatively good PS (PS 0:49.2%) and have tumors with Child‐Pugh Grade A (47.6%), minimal fibrosis (ISHAK score 0:30.5%), Grade 2 (47.6%), T3‐4 (32.3%), and TNM I stage (T1N0M0, 39.0%). The median age, BMI, tumor weight, serum AFP, ALB_adjust_, creatinine_adjust_, PLT, and PT of patients were 62, 25.1, 240, 13, −0.05, −0.05, 211, and 1.1, respectively, and the deaths occurred in the current cohort were 74 (45.1%).

### 3.2 Identification of six key lncRNAs for HCC patients' survival

**Figure 2:**
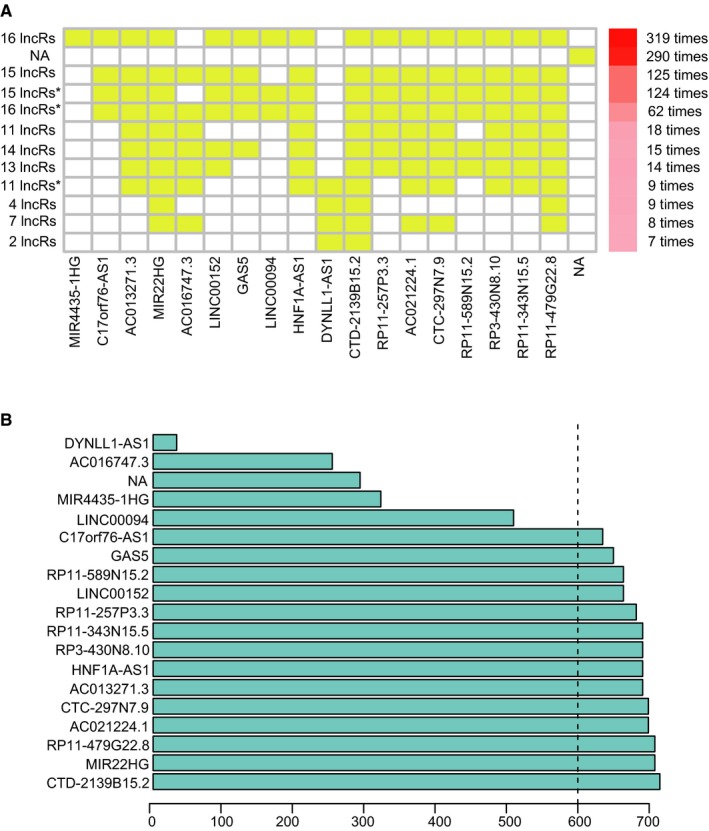
The seed lncRNAs were extracted by 1000 times Cox LASSO regression. A, Highly consistency was demonstrated in the lncRNAs among the 11 extracted lncRNA sets. The left ordinate indicates the seed lncRNA set and the number of seed lncRNAs found by every single iteration of LASSO. The right ordinate is the frequency of the seed lncRNA set disclosed through the 1000 times Cox LASSO regression. The horizontal ordinate is the lncRNA name. The yellow block represents the occurrence of the particular lncRNA in the specific lncRNA set; B, Totally 14 seed lncRNAs with >600 occurrences in the most common lncRNA set were filtered out for further analysis. The blue column indicates the frequency of each lncRNA occurs in the most common lncRNA set

Totally, 12 727 lncRNAs were analyzed for the prognostic significance in univariate Cox survival analysis, and 30 lncRNAs with *P* < 0.01 were filtered out and applied to 1000 times Cox LASSO regression with 10‐fold CV. As shown in Figure 2A, totally 11 lncRNA groups were disclosed, and the highly consistency among the lncRNA sets was demonstrated. In the most common lncRNA set, 14 lncRNAs were uncovered to have >600 occurrences and extracted for further analysis (Figure 2B). The multivariate Cox analysis with 1000 times bootstrapping based on the 14 lncRNAs finally identified six lncRNAs, including MIR22HG, CTC‐297N7.9, CTD‐2139B15.2, RP11‐589N15.2, RP11‐343N15.5, and RP11‐479G22.8, to be the independent predictors of patients' survival in HCC. The detailed information and the survival significance of the six lncRNAs are shown in Tables 1 and 2.

**Table 1:** Descriptions of the six lncRNAs

Hg19_name	Lnc ID	Ensembl_ID	Havana_gene	Gene_type	Chr	Start	End	Strand
MIR22HG	MIR22HG	ENSG00000186594.8	OTTHUMG00000132197.5	lincRNA	chr17	1614805	1620468	−
CTD‐2139B15.2	lnc‐BASP1‐1	ENSG00000248223.1	OTTHUMG00000161870.1	lincRNA	chr5	17354019	17355008	+
CTC‐297N7.9	lnc‐TMEM220‐1	ENSG00000264016.2	OTTHUMG00000178048.3	lincRNA	chr17	10644584	10672333	−
RP11‐589N15.2	lnc‐DEFB136‐2	ENSG00000269899.1	OTTHUMG00000183972.1	sense_intronic	chr8	11703663	11703900	−
RP11‐343N15.5	lnc‐FCGR1B‐12	ENSG00000269996.1	OTTHUMG00000184096.1	lincRNA	chr1	121133256	121134581	+
RP11‐479G22.8	lnc‐ITGB1‐1	ENSG00000273038.1	OTTHUMG00000186205.1	lincRNA	chr10	33176189	33178239	−

**Table 2:** The correlations of the six lncRNAs with patients' overall survival in HCC based on TCGA dataset using uni‐ and multivariate Cox analysis

Gene	Univariate Cox		Multivariate Cox		
	HR (95% CI)	*P*‐value	HR (95% CI)	*P*‐value	Bootstrapping 95% CI
MIR22HG	0.80 (0.68‐0.94)	0.0069	0.79 (0.68‐0.92)	0.0023	0.65‐0.91
CTD‐2139B15.2	1.06 (1.03‐1.09)	<0.0001	1.05 (1.02‐1.08)	0.0022	1.02‐1.10
CTC‐297N7.9	0.72 (0.58‐0.90)	0.0032	0.73 (0.58‐0.93)	0.0101	0.58‐0.94
RP11‐589N15.2	1.15 (1.04‐1.27)	0.0059	1.16 (1.03‐1.31)	0.0166	1.05‐1.32
RP11‐343N15.5	1.09 (1.03‐1.16)	0.0047	1.10 (1.03‐1.17)	0.0065	1.03‐1.18
RP11‐479G22.8	1.18 (1.09‐1.28)	<0.0001	1.12 (1.03‐1.23)	0.0119	1.03‐1.30

### 3.3 Confirmation for the prognostic role of the six‐lncRNA model for HCC

**Figure 3:**
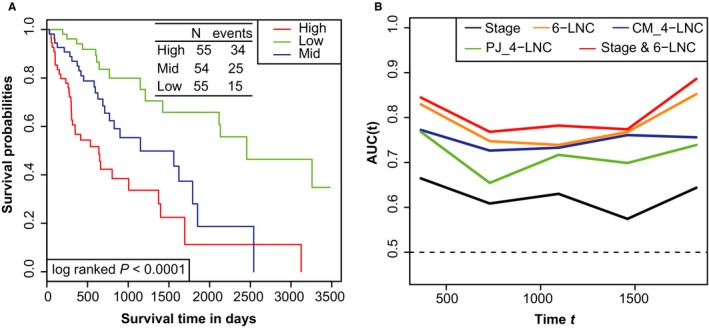
The prognostic significance and superiority of the novel six‐lncRNA signature were, respectively, illustrated by the Kaplan‐Meier (K‐M) analysis and the time‐dependent ROC curve analysis. A, K‐M analysis identified the prognosis significance of the six‐lncRNA signature for OS of patients in HCC. B, The six‐lncRNA signature was not only superior to the two published four‐lncRNA groups, but also increased the prediction accuracy of traditional prognostic TNM stage for OS. 6‐LNC: our six‐lncRNA model; CM_4‐LNC: the four‐lncRNA group published in Cancer Medicine; PJ_4‐LNC: the four‐lncRNA signature disclosed in Peer Journal; Stage and 6‐LNC: the prognostic model established based on both our six‐lncRNA signature and TNM stage

According to the K‐M analysis (Figure 3A), there is a significant difference in patients' survival among high‐, mid‐, and low‐risk groups divided by the six‐lncRNA signature (log‐rank test *P* < 0.0001), and patients in the high‐risk group had significantly shorter survival (median 21.3 months) than those in the mid‐ (median 38.3 months) and low‐risk groups (median 81.9 months).

In the time‐dependent ROC curve analysis (Figure 3B), the AUCs in the first, third, and fifth year are 0.830, 0.739, and 0.852, respectively, and the prediction capability of our six‐lncRNA signature is superior to the two published four‐lncRNA groups. Moreover, the inclusion of six‐lncRNA linear predictor to the prognostic model using TNM stage obviously improved the prediction ability for survival, as demonstrated by the increase of the resulting AUC values.

**Figure 4:**
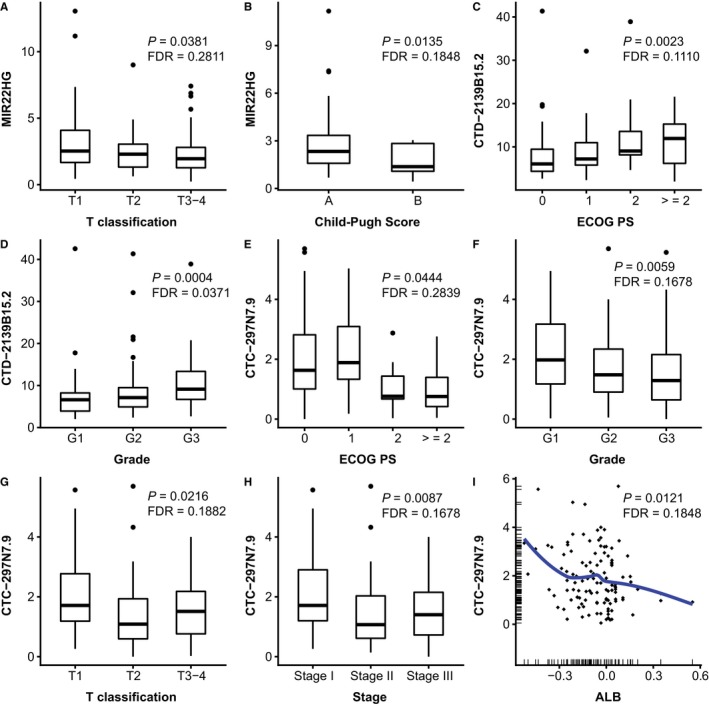
The significant trends in the associations of MIR22HG, CTD‐2139B15.2, and CTC‐297N7.9 expression with clinicopathological characteristics of the cohort. Low level of MIR22HG significantly indicated advanced T classification (A) and higher Child‐Pugh score (B) of HCC patients. Higher CTD‐2139B15.2 predicted higher ECOG PS score (C) and tumor grade (D). CTC‐297N7.9 was associated with low ECOG PS score of patients (E), the low grade (F), and early T classification (G) and TNM stage (H) of tumors, and the low serum levels of ALB (I). For categorical variables, the data distributions of each lncRNA were illustrated by the box plots. In the plot, the upper and lower hinge and the inner line of the boxes indicate the first and third quartile and the median value of the data, respectively, and the black dots represent the outlier values. For continuous variables, we used scatter plot with trend line illustrating their relationships with the level of each lncRNA

**Figure 5:**
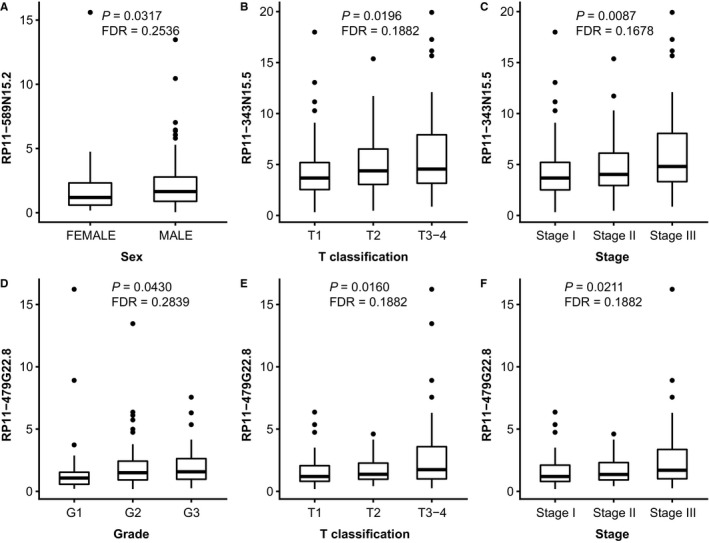
The analysis for the significant trends in the relationships between the levels of RP11‐589N15.2, RP11‐343N15.5, and RP11‐479G22.8 and the clinicopathological parameters of the HCC cohort. RP11‐589N15.2 was higher in male patients (A). RP11‐343N15.5 significantly predicted advanced T classification (B) and TNM stage (C) of HCC. Patients with advanced tumor grade (D), T classification (E), and TNM stage (F) expressed higher RP11‐479G22.8. For categorical variables, the data distributions of each lncRNA were illustrated by the box plots. In the plot, the upper and lower hinge and the inner line of the boxes indicate the first and third quartile and the median value of the data, respectively, and the black dots represent the outlier values.

The significant trends in the associations of each lncRNA expression with clinicopathological characteristics of the cohort are shown in Table 3 and Figures 4 and 5. On the one hand, we found two prognosis benefit lncRNAs. MIR22HG was demonstrated to be negatively correlated with T classification (*P* = 0.0381) and Child‐Pugh score (*P* = 0.0135) of HCC (Figure 4A,B), and CTC‐297N7.9 was negatively associated with ECOG PS of patients (*P* = 0.0444), the grade (*P* = 0.0059), T classification (*P* = 0.0216), and TNM stage (*P* = 0.0087) of tumors, and the serum levels of ALB (*P* = 0.0121) (Figure 4E‐I). On the other hand, four lncRNAs majorly indicated malignant phenotypes in HCC. CTD‐2139B15.2 was positively related with ECOG PS (*P* = 0.0023) and grade (*P* = 0.0004) (Figure 4C and D). RP11‐589N15.2 was observed higher in male patients (*P* = 0.0317) (Figure 5A), and RP11‐343N15.5 significantly predicted advanced T classification (*P* = 0.0196) and TNM stage of HCC (*P* = 0.0087) (Figure 5B,C). The significant positive relationships were disclosed between RP11‐479G22.8 levels and the tumor grade (*P* = 0.0430), T classification (*P* = 0.0160), and TNM stage (*P* = 0.0211) (Figure 5D‐F).

**Table 3:** The relationships of the 6 lncRNA level with the CPPs of 164 patients in TCGA

Variable	N (%)	MIR22HG		CTD‐2139B15.2		CTC‐297N7.9		RP11‐589N15.2		RP11‐343N15.5		RP11‐479G22.8	
		Med (IQR)	*P *(FDR)	Med (IQR)	*P *(FDR)	Med (IQR)	*P *(FDR)	Med (IQR)	*P *(FDR)	Med (IQR)	*P*	Med (IQR)	*P* (FDR)
Sex			—		—		—		0.0317 (0.2536)		—		—
Female	56 (34.1)	—		—		—		1.88 (0.59‐2.32)		—		—	
Male	108 (65.9)	—		—		—		1.65 (0.89‐2.78)		—		—	
PS			—		0.0023 (0.1110)		0.0444 (0.2839)		—		—		—
0	60 (49.2)	—		6.06 (4.36‐9.46)		1.63 (1.01‐2.82)		—		—		—	
1	39 (32.0)	—		7.20 (5.80‐10.98)		1.89 (1.33‐3.09)		—		—		—	
2	15 (12.3)	—		9.05 (8.16‐13.57)		0.76 (0.67‐1.44)		—		—		—	
≥2	8 (6.6)	—		11.94 (6.19‐15.26)		0.75 (0.42‐1.39)		—		—		—	
CPG			0.0135 (0.1848)		—		—		—		—		—
A	78 (47.6)	2.33 (1.58‐3.34)		—		—		—		—		—	
B	17 (10.4)	1.37 (1.08‐2.83)		—		—		—		—		—	
Grade			—		0.0004 (0.0371)		0.0059 (0.1678)		—		—		0.0430 (0.2839)
1	32 (19.5)	—		6.61 (3.91‐8.23)		1.98 (1.17‐3.17)		—		—		1.07 (0.57 ‐ 1.54)	
2	78 (47.6)	—		7.11 (4.91‐9.49)		1.48 (0.90‐2.34)		—		—		1.50 (0.92‐2.43)	
3	50 (30.5)	—		9.12 (6.69‐13.35)		1.29 (0.64‐2.15)		—		—		1.57 (0.97‐2.63)	
T stage			0.0381 (0.2811)		—		0.0216 (0.1882)		—		0.0196 (0.1882)		0.0160 (0.1882)
T1	2 (1.2)	2.52 (1.67‐4.09)		—		1.71 (1.19‐2.77)		—		3.67 (2.54‐5.19)		1.19 (0.81‐2.06)	
T2	43 (26.2)	2.29 (1.32‐3.04)		—		1.09 (0.60‐1.93)		—		4.38 (3.04‐6.52)		1.37 (0.97‐2.27)	
T3‐4	53 (32.3)	1.95 (1.27‐2.80)		—		1.51 (0.76‐2.18)		—		4.55 (3.16‐7.92)		1.74 (1.00‐3.59)	
Stage			—		—		0.0087 (0.1678)		—		0.0087 (0.1678)		0.0211 (0.1882)
I	64 (39.0)	—		—		1.71 (1.20‐2.90)		—		3.67 (2.51‐5.21)		1.19 (0.80‐2.11)	
II	40 (24.4)	—		—		1.07 (0.61‐2.03)		—		4.02 (2.94‐6.11)		1.36 (0.92‐2.31)	
III	51 (31.1)	—		—		1.40 (0.73‐2.15)		—		4.80 (3.31‐8.05)		1.70 (1.01‐3.36)	
ALB		—	—	—	—	−0.05 (−0.18‐0.03)	0.0121 (0.1848)	—	—	—	—	—	—

CPG, Child‐Pugh grade; CPPs, clinicopathological characteristics; IQR, interquartile range; Med, median; PS, performance status.

### 3.4 Elucidation for the functional roles of the six key lncRNAs

**Figure 6:**
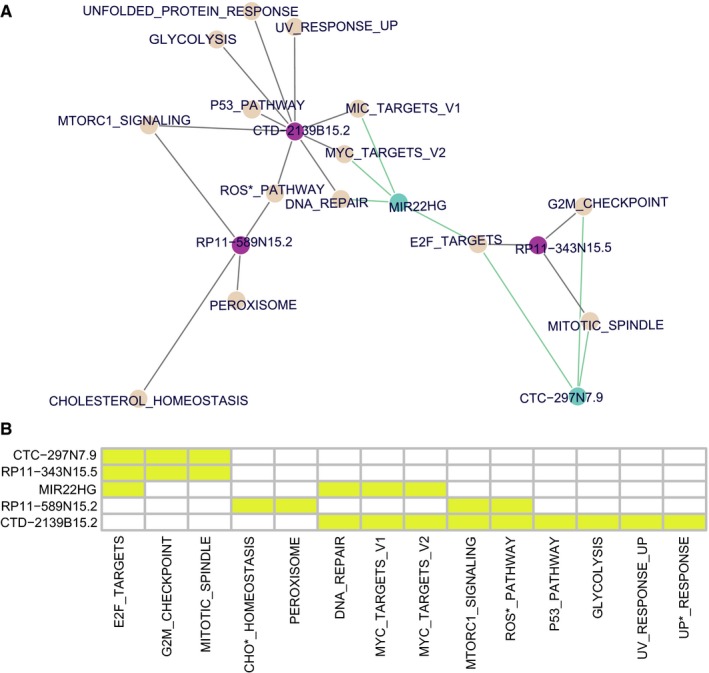
Gene set enrichment analysis for the six prognostic lncRNAs screened out multiple hallmark gene sets related with cell cycle, DNA repair, cell homeostasis, or stress response. The network (A) and the heat map (B) illustrate the significant gene sets enriched by the six lncRNAs. In the network (A), the brown nodes represent the enriched hallmark gene sets, the purple nodes represent the lncRNAs related with poorer prognosis, and the green nodes represent the lncRNAs related with better prognosis of patients. In the heat map (B), the yellow block indicates the particular hallmark gene set enriched by the specific lncRNA

For each lncRNA, multiple significant gene sets were extracted by GSEA based on FDR q‐values (Table 4) and illustrated by both network and heat map (Figure 6A and B). Low levels of MIR22HG and CTC‐297N7.9, but high levels of RP11‐343N15.5, were significantly enriched with cell cycle or DNA repair‐related gene sets, such as E2F_TARGETS, G2M_CHECKPOINT, MITOTIC_SPINDLE, DNA_REPAIR, MYC_TARGETS. However, high expressions of CTD‐2139B15.2 and RP11‐589N15.2 were found to enrich the gene sets related with cell homeostasis or stress response, such as P53_PATHWAY, MITORC1_SIGNALING, MYC_TARGETS_V1 and _V2, UNFOLDED_PROTEIN_REPONSE, REACTIVE_OXIGEN_SPECIES_PATHWAY, UV_RESPONSE_UP, and so on.

**Table 4:** Gene set enrichment analysis and leading‐edge gene assays according to the levels of the 6 lncRNAs in TCGA

		Size	ES	*P*	FDR.q.	Rank_max_	Leading edge
MIR22HG	DNA_REPAIR	149	−0.51	0.0043	0.1123	3741	tags = 48%, list = 18%, signal = 59%
	MYC_TARGETS_V1	195	−0.59	0.0142	0.0791	3847	tags = 54%, list = 19%, signal = 66%
	E2F_TARGETS	193	−0.68	0.0122	0.0527	2780	tags = 56%, list = 14%, signal = 64%
	MYC_TARGETS_V2	58	−0.62	0.0354	0.1012	4306	tags = 60%, list = 21%, signal = 76%
CTD‐2139B15.2	MITORC1_SIGNALING	198	0.56	<0.0001	0.0039	3572	tags = 46%, list = 17%, signal = 56%
	UV_RESPONSE_UP	158	0.45	0.0017	0.0717	4141	tags = 41%, list = 20%, signal = 50%
	MYC_TARGETS_V1	195	0.65	0.0077	0.0711	3519	tags = 59%, list = 17%, signal = 70%
	UNFOLDED_PROTEIN_REPONSE	113	0.49	0.0037	0.0658	4531	tags = 49%, list = 22%, signal = 62%
	GLYCOLYSIS	199	0.44	0.0107	0.0615	3943	tags = 38%, list = 19%, signal = 46%
	MYC_TARGETS_V2	58	0.65	0.0277	0.1314	3324	tags = 60%, list = 16%, signal = 72%
	P53_PATHWAY	196	0.40	0.0086	0.1377	4259	tags = 38%, list = 21%, signal = 47%
	REACTIVE_OXIGEN_SPECIES_PATHWAY	48	0.55	0.0383	0.1401	4553	tags = 50%, list = 22%, signal = 64%
	DNA_REPAIR	149	0.46	0.0302	0.1549	5511	tags = 54%, list = 27%, signal = 73%
CTC‐297N7.9	E2F_TARGETS	193	−0.73	<0.0001	0.0106	3063	tags = 70%, list = 15%, signal = 81%
	G2M_CHECKPOINT	194	−0.70	0.0021	0.0057	3596	tags = 68%, list = 18%, signal = 81%
	MITOTIC_SPINDLE	198	−0.54	0.0022	0.0560	3759	tags = 42%, list = 18%, signal = 51%
RP11‐589 N15.2	CHOLESTEROL_HOMEOSTASIS	74	0.70	<0.0001	<0.0001	1266	tags = 43%, list = 6%, signal = 46%
	MITORC1_SIGNALING	198	0.55	0.0038	0.0140	3797	tags = 48%, list = 18%, signal = 59%
	REACTIVE_OXIGEN_SPECIES_PATHWAY	48	0.62	0.0115	0.0954	3769	tags = 60%, list = 18%, signal = 74%
	PEROXISOME	102	0.49	0.0218	0.1735	5318	tags = 56%, list = 26%, signal = 75%
RP11‐343N15.5	G2M_CHECKPOINT	194	0.69	0.0057	0.0746	4034	tags = 69%, list = 20%, signal = 85%
	MITOTIC_SPINDLE	198	0.54	0.0056	0.1026	4703	tags = 53%, list = 23%, signal = 67%
	E2F_TARGETS	193	0.70	0.0114	0.0854	3367	tags = 67%, list = 16%, signal = 79%

**Figure 7:**
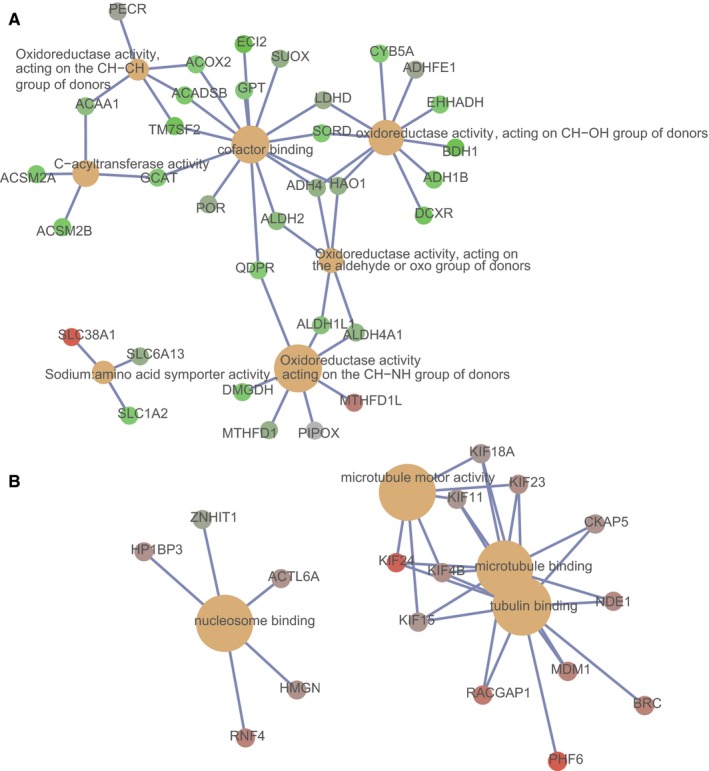
GO‐MF analysis for the coexpression mRNAs of RP11‐479G22.8 and RP11‐343N15.5. The cnetplot illustration was used to visualize the GO‐MF terms enriched by the coexpressed mRNAs of RP11‐479G22.8 (A) and RP11‐343N15.5 (B). The brown node represents the enriched GO‐MF term, with the size indicating the overall number of its included mRNAs. The other smaller nodes are the enriched mRNAs, and the node colors changing from green to red indicate the increased associations of the mRNAs with the specific lncRNA

In the Pearson correlation analysis of the key lncRNAs with mRNAs using TCGA profile, there were, respectively, 12, 784, 55, 8, 240, and 186 genes closely related with MIR22HG, CTC‐297N7.9, CTD‐2139B15.2, RP11‐589N15.2, RP11‐343N15.5, and RP11‐479G22.8. According to further GO‐MF analysis for the specific lncRNA‐related mRNAs, we supposed that RP11‐479G22.8 took part in the modifications of “oxidoreductase activity,” “cofactor binding,” and “C‐acyltransferase activity” (Figure 7A); RP11‐343N15.5 played key roles in tubulin/nucleosome/microtubule binding and “microtubule motor activity” (Figure 7B); CTD‐2139B15.2 potentially functioned in “ligase activity” and “magnesium ion binding” (Figure S1A); and CTC‐297N7.9 majorly involved in the cofactor/chromatin/NAD binding and the oxidoreductase/DNA‐dependent ATPase activity (Figure S1B). Detailed information of the top five GO‐MF terms based on *P* values is listed in Table 5.

**Table 5:** GO‐MF assays according to the levels of the six‐lncRNA‐related mRNAs in TCGA

	ID	Description	GeneRatio	BgRatio	*P*	*P* adjust	*Q*	Count
CTD‐2139B15.2^a^	GO:0004812	Aminoacyl‐tRNA ligase activity	4/46	44/16982	5.91E−06	0.00041374	0.0003102	4
	GO:0016875	Ligase activity, forming carbon‐oxygen bonds	4/46	44/16982	5.91E−06	0.00041374	0.0003102	4
	GO:0016876	Ligase activity, forming aminoacyl‐tRNA and related compounds	4/46	44/16982	5.91E−06	0.00041374	0.0003102	4
	GO:0016874	Ligase activity	6/46	177/16982	7.81E−06	0.00041374	0.0003102	6
	GO:0000287	Magnesium ion binding	5/46	196/16982	0.0001816	0.00770244	0.00577491	5
CTC‐297N7.9^a^	GO:0048037	Cofactor binding	50/714	264/16982	1.72E−19	1.26E−16	1.10E−16	50
	GO:0016614	Oxidoreductase activity, acting on CH‐OH group of donors	27/714	137/16982	1.55E−11	3.80E−09	3.29E−09	27
	GO:0003682	Chromatin binding	46/714	482/16982	1.91E−07	2.50E−05	2.16E−05	46
	GO:0051287	NAD binding	13/714	53/16982	2.04E−07	2.50E−05	2.16E−05	13
	GO:0008094	DNA‐dependent ATPase activity	15/714	76/16982	5.10E−07	5.34E−05	4.63E−05	15
RP11‐343N15.5	GO:0015631	Tubulin binding	12/214	289/16982	0.00031283	0.04698072	0.04389422	12
	GO:0031491	Nucleosome binding	5/214	49/16982	0.00036839	0.04698072	0.04389422	5
	GO:0003777	Microtubule motor activity	6/214	77/16982	0.00042041	0.04698072	0.04389422	6
	GO:0008017	Microtubule binding	10/214	217/16982	0.0004401	0.04698072	0.04389422	10
RP11‐479G22.8^a^	GO:0016645	Oxidoreductase activity, acting on the CH‐NH group of donors	7/170	28/16982	8.83E−09	3.72E−06	3.18E−06	7
	GO:0048037	Cofactor binding	14/170	264/16982	4.39E−07	9.24E−05	7.90E−05	14
	GO:0016614	Oxidoreductase activity, acting on CH‐OH group of donors	10/170	137/16982	1.19E−06	0.00012535	0.00010719	10
	GO:0016408	C‐acyltransferase activity	4/170	20/16982	4.14E−05	0.00249223	0.00213112	4
	GO:0016903	Oxidoreductase activity, acting on the aldehyde or oxo group of donors	5/170	45/16982	8.38E−05	0.00440768	0.00376904	5

a The first five GO‐MF terms based on *P* values were listed.

## 4 DISCUSSION

By applying multiple biostatistics methods, such as univariate Cox and 1000 times Cox LASSO regression with 10‐fold CV and multivariate Cox analysis with 1000 times bootstrapping, on the overall lncRNA data of appropriately selected cases in TCGA, six lncRNAs, MIR22HG, CTC‐297N7.9, CTD‐2139B15.2, RP11‐589N15.2, RP11‐343N15.5, and RP11‐479G22.8, were currently filtered out and identified as the independent prognosis predictors in HCCs. The prognostic significance, the prediction superiority, and the clinicopathological roles of the six‐lncRNA signature were, respectively, confirmed by K‐M analysis, time‐dependent ROC curves, and appropriate correlation analysis tools. Further GSEA, and the extraction and GO‐MF assays of functional‐related genes uncovered the regulation of cell cycle and stress response‐related pathways to be the vital functions of the six lncRNAs in HCC progression.

LncRNAs are considered to have greater potentiality than other hallmarks of cancers as the biomarkers of diagnosis and prognosis because of following unique advantages. (a) The expressions of lncRNAs show great divergence in different tissues, diseases and the disease progression stage, thus are more representative of disease characteristics.^26^, ^27^ (b) LncRNAs are noncoding RNAs and directly involve in various biological processes, thus the levels and functions are more closely associated with the development characteristics of diseases including cancers.^28^, ^29^, ^30^, ^31^ Therefore, more and more studies are performed to clarify the clinical significance of lncRNAs in cancers, including HCCs.

Although more and more lncRNAs have been identified involved in various diseases including cancers, the functions of most lncRNAs are still not well understood, and a large number of lncRNAs are awaiting further characterizations. Accordingly, it is popular to predict the functions of lncRNAs by GSEA, GO‐MF, and lncRNA‐mRNA coexpression analysis.^32^, ^33^ With these popular methods, the six lncRNAs were currently unraveled to potentially involve in multiple ontogenetic mechanisms, such as cell cycle, DNA repair, cell homeostasis, and stress response, via variety of functions including “ligase activity,” “magnesium ion binding,” cofactor/chromatin/NAD binding, oxidoreductase/DNA‐dependent ATPase activity, tubulin/nucleosome/microtubule binding, “microtubule motor activity,” and “C‐acyltransferase activity,” which were regarded fundamental for transcription regulation and chromatin modification, and important for HCC development and progression.^2^, ^30^, ^31^

On the one hand, MIR22HG and CTC‐297N7.9 are found to predict better prognosis of HCCs among the six lncRNAs, and these results could be well supported by their negative relationships with the advanced CPPs revealed in the current study and are consistent with the previous studies of MIR22HG in HCCs and lung adenocarcinomas and CTC‐297N7.9 in HCCs.^13^, ^34^, ^35^ Functionally, we demonstrate the involvement of MIR22HG in cell cycle and DNA repair pathways, and thus present further bioinformatics evidence for the recent observations that MIR22HG prohibits the proliferation of liver cancer cells and inhibits cell cycle‐related genes via the regulation of YBX1, Met, and P21 in lung cancer.^35^, ^36^ Previous reports showed that CTC‐297N7.9 prohibited cancer development via the transmembrane protein, and our current study discloses that CTC‐297N7.9 negatively regulates the pathways of E2F_TARGETS, G2M_CHECKPOINT, and MITOTIC_SPINDLE via involving in cofactor/chromatin/NAD binding and oxidoreductase/DNA‐dependent ATPase activity.

On the other hand, the other four lncRNAs, including CTD‐2139B15.2, RP11‐589N15.2, RP11‐343N15.5, and RP11‐479G22.8, are currently demonstrated to be poorer prognosis indicators for HCCs, and the reports on the prognostic roles of CTD‐2139B15.2 in papillary thyroid cancer^37^ and RP11‐479G22.8 in lung adenocarcinoma^38^ tally with the findings, whereas there is no previous report on the clinicopathological relevance of RP11‐589N15.2 and RP11‐343N15.5 in cancers. Moreover, the harmful roles of the four lncRNAs in HCCs are presently further illustrated by their positive relationships with the progressive CPPs. So far, the functional engagement of the four lncRNAs in cancers is unknown. For the first time, we uncovered the involvement of CTD‐2139B15.2 in cell homeostasis and stress response through “ligase activity” and “magnesium ion binding” and RP11‐343N15.5 in cell cycle progression via tubulin/nucleosome/microtubule binding and “microtubule motor activity,” and disclosed the roles of RP11‐589N15.2 in cell homeostasis and stress response and the “oxidoreductase activity,” “cofactor binding,” and “C‐acyltransferase activity” of RP11‐479G22.8 in HCCs. As for the molecular functions of RP11‐589N15.2 and the biological processes that involved by RP11‐479G22.8, there are no statistical findings in the present study based on the current dataset, and more biostatistics analysis and molecular studies are needed.

Comparing with other studies for the prognostic roles of lncRNAs in HCCs, our study exhibits several superiorities. In the initial step, it is more sensible to identify prognostic molecules from global lncRNAs, rather than starting from differential expression ones. On the study cohort, homogeneity of patients is greatly improved by excluding the specific cases might have distinct disease procedures, such as those diagnosed as cholangiocarcinoma, received neoadjuvant therapy, survived too short durations, and had distant metastasis or residual tumors. On the study method, the accuracy of the bioinformatics analysis is increased by integrating 1000 times LASSO regression and bootstrapping, and the clinical reasonability of the biomarker study is validated by the functional analysis combining with the CPPs of cancers and the physical status of patients. As for the prediction efficacy, our six‐lncRNA signature was helpful to improve the prediction accuracy of traditional prognostic TNM stage and showed superiority than the two revealed four‐lncRNA groups^13^, ^15^ based on the increase of the resulting AUC values in the analysis of the ROC curves. Additionally, the lncRNA SNHG20 and SERHL disclosed by Ma et al in the recently published four‐lncRNA‐signature study^17^ were demonstrated prognostic insignificant (*P* = 0.1072 and 0.1304, respectively, in univariate Cox analysis) in the current cohort of patients carefully selected based on the clinicopathological characteristics.

Several limitations should be considered. First, we did not perform in vitro and in vivo experimental studies to confirm the prognostic role of the six‐lncRNA signature in HCCs, which was deduced from online datasets using bioinformatics methods. Second, there is no other available dataset so far that could be used to present more external validations for the results of the current study. Further validations are awaited.

In summary, we uncover a six‐lncRNA signature as the independent prognosis biomarker in HCCs by the comprehensive bioinformatics analysis combining the genetic profile and CPPs data in carefully selected cohort, confirm its’ superior capability for resectable HCC prognosis prediction based on the ROC curves comparing with the other multiple‐lncRNA combinations and the traditional CPPs, and reveal the regulation of cell cycle and stress response‐related pathways via transcription and chromatin modifications as the potential functional roles of the six lncRNAs in resectable HCC progression. The results not only disclose the novel candidate biomarker for HCC outcome prediction, but also indicate the interesting topics for future studies on the underlying mechanisms of HCC progression and the potential functions of the six lncRNAs.

## CONFLICT OF INTEREST

None declared.
