# Supplementary material for: Genomewide identification of a novel six‐LncRNA signature to improve prognosis prediction in resectable hepatocellular carcinoma
Source: Cancer Med. 2018 Oct 30;7(12):6219–33. doi: 10.1002/cam4.1854 (PMC6308084; doi:10.1002/cam4.1854)
Supplement: Supplementary file 2 [file CAM4-7-6219-s002.docx]

Supplementary Table 1. Basic characteristics of patients in TCGA cohort

|  | N (%) |  |  | N - NA | Median (IQR) |
| --- | --- | --- | --- | --- | --- |
| Sex |  |  | Age | 0 | 62 (53 - 70) |
| Female | 56 (34.1) |  | BMI | 14 | 25.1 (21.5 - 29.7) |
| Male | 108 (65.9) |  | Tumor weight | 12 | 240 (140 - 452.5) |
| Performance Status |  |  | AFP | 49 | 13 (4 - 228) |
| 0 | 60 (49.2) |  | ALB | 48 | -0.05 (-0.18 - 0.03) |
| 1 | 39 (32.0) |  | Creatinine | 45 | -0.05 (-0.2 - 0.16) |
| 2 | 15 (12.3) |  | PLT | 37 | 211 (155 – 282.5) |
| >= 2 | 8 (6.6) |  | PT | 42 | 1.1 (1 – 10.18) |
| Risk factor |  |  |  |  |  |
| Alcohol consumption | 47 (28.7) |  |  |  |  |
| Hepatitis virus | 38 (23.2) |  |  |  |  |
| Others | 31 (18.9) |  |  |  |  |
| None | 48 (29.3) |  |  |  |  |
| Child-Pugh grade |  |  |  |  |  |
| NA | 69 (42.1) |  |  |  |  |
| A | 78 (47.6) |  |  |  |  |
| B | 17 (10.4) |  |  |  |  |
| ISHAK score |  |  |  |  |  |
| NA | 66 (40.2) |  |  |  |  |
| 0 | 50 (30.5) |  |  |  |  |
| 1-4 | 22 (13.4) |  |  |  |  |
| 5-6 | 26 (15.9) |  |  |  |  |
| Grade |  |  |  |  |  |
| NA | 4 (2.4) |  |  |  |  |
| 1 | 32 (19.5) |  |  |  |  |
| 2 | 78 (47.6) |  |  |  |  |
| 3 | 50 (30.5) |  |  |  |  |
| T classification |  |  |  |  |  |
| NA | 2 (1.2) |  |  |  |  |
| T1 | 2 (1.2) |  |  |  |  |
| T2 | 43 (26.2) |  |  |  |  |
| T3 - 4 | 53 (32.3) |  |  |  |  |
| TNM stage |  |  |  |  |  |
| NA | 9 (5.5) |  |  |  |  |
| I | 64 (39.0) |  |  |  |  |
| II | 40 (24.4) |  |  |  |  |
| III | 51 (31.1) |  |  |  |  |
| IQR, interquartile range; NA, not available / unknown | | | | | |

| Supplementary Table 2. The major clinicopathological data of 164 HCC patients in the current analysis | | | | | | |  |  |  |
| --- | --- | --- | --- | --- | --- | --- | --- | --- | --- |
| Sample ID | OS | OS-event | Sex | Age | ECOG PS | Grade | T classification | N classification | Stage |
| TCGA-BC-A10R | 308 | 1 | FEMALE | 66 | NA | G2 | T3-4 | NX | NA |
| TCGA-BC-A10S | 1423 | 1 | MALE | 81 | NA | G1 | T3-4 | NX | NA |
| TCGA-BC-A10X | 770 | 1 | FEMALE | 52 | NA | G2 | T3-4 | N0 | Stage III |
| TCGA-BC-A10Y | 711 | 1 | MALE | 76 | NA | G3 | T3-4 | NX | NA |
| TCGA-BC-A10Z | 34 | 1 | FEMALE | 62 | NA | G2 | T1 | N0 | Stage I |
| TCGA-BC-A110 | 2116 | 1 | FEMALE | 51 | NA | G1 | T1 | NX | NA |
| TCGA-BC-A112 | 153 | 1 | MALE | 80 | NA | G2 | T3-4 | NX | NA |
| TCGA-BC-A216 | 1351 | 0 | FEMALE | 62 | NA | G2 | T3-4 | NX | Stage III |
| TCGA-BC-A217 | 1397 | 1 | FEMALE | 75 | NA | G3 | T2 | NX | Stage II |
| TCGA-BC-A3KG | 680 | 0 | FEMALE | 68 | NA | G3 | T2 | N0 | Stage II |
| TCGA-BC-A69H | 444 | 0 | MALE | 64 | 1 | G3 | T2 | NX | Stage II |
| TCGA-BC-A69I | 387 | 0 | MALE | 69 | 0 | G1 | T1 | N0 | Stage I |
| TCGA-BD-A2L6 | 1363 | 0 | MALE | 69 | 1 | G2 | T2 | NX | NA |
| TCGA-BD-A3EP | 409 | 0 | FEMALE | 75 | 1 | G2 | T1 | N0 | Stage I |
| TCGA-BD-A3ER | 1115 | 0 | MALE | 62 | 0 | G2 | T2 | NX | Stage II |
| TCGA-CC-5258 | 129 | 1 | MALE | 48 | above_2 | G2 | T2 | N0 | Stage II |
| TCGA-CC-5259 | 250 | 0 | FEMALE | 60 | NA | G2 | T3-4 | N0 | Stage III |
| TCGA-CC-5260 | 87 | 1 | FEMALE | 61 | NA | G1 | T3-4 | N0 | Stage III |
| TCGA-CC-5261 | 97 | 1 | MALE | 44 | NA | G2 | T2 | N0 | Stage II |
| TCGA-CC-5262 | 103 | 1 | MALE | 67 | NA | G1 | T3-4 | N0 | Stage III |
| TCGA-CC-5263 | 129 | 1 | MALE | 35 | NA | G1 | T3-4 | N0 | Stage III |
| TCGA-CC-5264 | 102 | 1 | MALE | 71 | NA | G2 | T3-4 | N0 | Stage III |
| TCGA-CC-A1HT | 101 | 1 | MALE | 50 | above_2 | G3 | T3-4 | N0 | Stage III |
| TCGA-CC-A3M9 | 300 | 1 | MALE | 45 | 2 | G3 | T3-4 | N0 | Stage III |
| TCGA-CC-A3MA | 303 | 1 | MALE | 61 | above_2 | G2 | T3-4 | N0 | Stage III |
| TCGA-CC-A3MB | 315 | 1 | MALE | 36 | above_2 | G1 | T3-4 | N0 | Stage III |
| TCGA-CC-A3MC | 363 | 0 | MALE | 54 | above_2 | G2 | T3-4 | N0 | Stage III |
| TCGA-CC-A5UC | 347 | 1 | MALE | 63 | 2 | G3 | T3-4 | N0 | Stage III |
| TCGA-CC-A5UD | 304 | 1 | MALE | 45 | 1 | G2 | T3-4 | N0 | Stage III |
| TCGA-CC-A5UE | 272 | 1 | MALE | 48 | above_2 | G2 | T3-4 | N0 | Stage III |
| TCGA-CC-A7IF | 649 | 1 | MALE | 59 | above_2 | G1 | T3-4 | N0 | Stage III |
| TCGA-CC-A7IG | 299 | 1 | MALE | 47 | 2 | G2 | T2 | N0 | Stage II |
| TCGA-CC-A7IH | 365 | 0 | MALE | 58 | above_2 | G1 | T3-4 | N0 | Stage III |
| TCGA-CC-A7II | 399 | 0 | MALE | 54 | 2 | G3 | T3-4 | N0 | Stage III |
| TCGA-CC-A7IJ | 382 | 0 | MALE | 56 | 2 | G3 | T2 | N0 | Stage II |
| TCGA-CC-A7IK | 262 | 1 | MALE | 59 | 2 | G3 | T3-4 | N0 | Stage III |
| TCGA-CC-A7IL | 278 | 1 | MALE | 61 | 2 | G1 | T3-4 | N0 | Stage III |
| TCGA-DD-A113 | 2425 | 0 | FEMALE | 55 | 1 | G3 | T2 | N0 | Stage II |
| TCGA-DD-A114 | 1149 | 1 | MALE | 42 | 1 | G3 | T2 | NA | Stage II |
| TCGA-DD-A115 | 2542 | 1 | MALE | 53 | 0 | G2 | T3-4 | N0 | Stage III |
| TCGA-DD-A116 | 1622 | 1 | MALE | 68 | 1 | G3 | T3-4 | N0 | Stage III |
| TCGA-DD-A118 | 3437 | 0 | FEMALE | 77 | 1 | G2 | T2 | N0 | Stage II |
| TCGA-DD-A11A | 79 | 0 | MALE | 67 | 0 | G3 | T1 | N0 | Stage I |
| TCGA-DD-A11C | 662 | 0 | MALE | 69 | 1 | G3 | T1 | N0 | Stage I |
| TCGA-DD-A11D | 1560 | 1 | FEMALE | 57 | 1 | G2 | T1 | N0 | Stage I |
| TCGA-DD-A1EA | 2415 | 0 | MALE | 68 | 0 | G2 | T2 | N0 | Stage II |
| TCGA-DD-A1EB | 2017 | 0 | FEMALE | 72 | 1 | G2 | T1 | N0 | Stage I |
| TCGA-DD-A1EC | 602 | 0 | FEMALE | 20 | 1 | G3 | T1 | N0 | Stage I |
| TCGA-DD-A1ED | 2301 | 0 | MALE | 68 | 1 | G1 | T1 | N0 | Stage I |
| TCGA-DD-A1EE | 349 | 1 | MALE | 73 | 2 | G3 | T3-4 | N0 | Stage III |
| TCGA-DD-A1EF | 394 | 1 | FEMALE | 57 | 2 | G3 | T1 | N0 | Stage I |
| TCGA-DD-A1EG | 1372 | 1 | MALE | 76 | 0 | G3 | T1 | N0 | Stage I |
| TCGA-DD-A1EH | 1495 | 0 | MALE | 23 | 1 | G3 | T3-4 | N0 | Stage III |
| TCGA-DD-A1EI | 183 | 0 | MALE | 46 | 2 | G2 | T1 | N0 | Stage I |
| TCGA-DD-A1EJ | 1005 | 1 | FEMALE | 71 | 2 | G2 | T1 | N1 | Stage III |
| TCGA-DD-A1EL | 415 | 1 | MALE | 23 | 0 | G3 | T2 | N0 | Stage II |
| TCGA-DD-A39V | 643 | 1 | MALE | 77 | 1 | G3 | T2 | NX | Stage II |
| TCGA-DD-A39W | 827 | 1 | FEMALE | 29 | NA | G2 | T3-4 | N0 | Stage III |
| TCGA-DD-A39X | 1694 | 1 | FEMALE | 78 | 1 | G2 | T1 | NX | Stage I |
| TCGA-DD-A39Y | 171 | 1 | MALE | 67 | 2 | G3 | T1 | NX | Stage I |
| TCGA-DD-A39Z | 601 | 1 | FEMALE | 43 | 2 | G2 | T2 | NX | Stage II |
| TCGA-DD-A3A1 | 233 | 1 | MALE | 65 | 0 | G2 | T3-4 | N0 | Stage III |
| TCGA-DD-A3A2 | 2131 | 1 | FEMALE | 76 | 0 | G1 | T1 | N0 | Stage I |
| TCGA-DD-A3A3 | 535 | 1 | MALE | 45 | 1 | G2 | T1 | N0 | Stage I |
| TCGA-DD-A3A4 | 612 | 1 | MALE | 37 | 0 | G3 | T3-4 | N0 | Stage III |
| TCGA-DD-A3A5 | 3125 | 1 | FEMALE | 66 | 1 | G2 | T3-4 | N0 | Stage III |
| TCGA-DD-A3A6 | 3258 | 1 | FEMALE | 72 | 0 | G2 | T2 | N0 | Stage II |
| TCGA-DD-A3A7 | 419 | 1 | MALE | 67 | 1 | G3 | T3-4 | N0 | Stage III |
| TCGA-DD-A4NA | 1008 | 0 | FEMALE | 67 | 0 | G3 | T2 | N1 | Stage III |
| TCGA-DD-A4ND | 2746 | 0 | FEMALE | 56 | 1 | G3 | T1 | N0 | Stage I |
| TCGA-DD-A4NE | 660 | 1 | FEMALE | 75 | 0 | G3 | T3-4 | N0 | Stage III |
| TCGA-DD-A4NF | 942 | 0 | MALE | 72 | 0 | G2 | T1 | N0 | Stage I |
| TCGA-DD-A4NG | 802 | 1 | MALE | 77 | 1 | G2 | T3-4 | NX | Stage III |
| TCGA-DD-A4NH | 917 | 0 | FEMALE | 65 | 0 | G3 | T3-4 | N0 | Stage III |
| TCGA-DD-A4NI | 816 | 0 | MALE | 67 | 0 | G2 | T2 | NX | Stage II |
| TCGA-DD-A4NJ | 928 | 0 | FEMALE | 54 | 0 | G2 | T2 | N0 | Stage II |
| TCGA-DD-A4NK | 1210 | 1 | FEMALE | 80 | 0 | G2 | T3-4 | N0 | Stage III |
| TCGA-DD-A4NL | 1711 | 0 | MALE | 46 | 1 | G1 | T1 | N0 | Stage I |
| TCGA-DD-A4NN | 899 | 1 | FEMALE | 56 | 0 | G3 | T1 | N0 | Stage I |
| TCGA-DD-A4NO | 2245 | 0 | MALE | 65 | 0 | G1 | T1 | N0 | Stage I |
| TCGA-DD-A4NP | 3308 | 0 | MALE | 32 | 0 | G3 | T1 | N0 | Stage I |
| TCGA-DD-A4NQ | 373 | 1 | MALE | 60 | 0 | G3 | T2 | N0 | Stage II |
| TCGA-DD-A4NS | 2456 | 1 | FEMALE | 61 | 0 | G2 | T1 | N0 | Stage I |
| TCGA-DD-A4NV | 2398 | 0 | MALE | 61 | 1 | G1 | T3-4 | N0 | Stage III |
| TCGA-DD-A73A | 728 | 0 | MALE | 71 | 1 | G2 | T1 | N0 | Stage I |
| TCGA-DD-A73B | 283 | 1 | FEMALE | 72 | 0 | G2 | T1 | N0 | Stage I |
| TCGA-DD-A73C | 701 | 0 | FEMALE | 65 | 0 | G1 | T3-4 | N0 | Stage III |
| TCGA-DD-A73D | 693 | 0 | FEMALE | 68 | 0 | G1 | T2 | NX | Stage II |
| TCGA-DD-A73E | 44 | 0 | MALE | 66 | 0 | G1 | T1 | N0 | Stage I |
| TCGA-DD-A73F | 1085 | 0 | FEMALE | 77 | 1 | G1 | T1 | N0 | Stage I |
| TCGA-DD-A73G | 3478 | 0 | FEMALE | 73 | 0 | G3 | T1 | N0 | Stage I |
| TCGA-ED-A459 | 910 | 0 | MALE | 47 | NA | G2 | T2 | N0 | Stage II |
| TCGA-ED-A4XI | 819 | 0 | MALE | 58 | NA | G3 | T2 | N0 | Stage II |
| TCGA-ED-A5KG | 854 | 0 | FEMALE | 60 | NA | G2 | T2 | N0 | Stage II |
| TCGA-ED-A627 | 423 | 0 | MALE | 74 | 2 | G2 | T1 | NX | Stage I |
| TCGA-ED-A66X | 406 | 0 | MALE | 35 | NA | G3 | T3-4 | N0 | Stage III |
| TCGA-ED-A66Y | 296 | 1 | FEMALE | 51 | NA | G3 | T3-4 | N0 | Stage III |
| TCGA-ED-A7PY | 390 | 0 | FEMALE | 20 | NA | G3 | T2 | NX | Stage II |
| TCGA-ED-A7XP | 400 | 0 | FEMALE | 53 | NA | G3 | T2 | N0 | Stage II |
| TCGA-ED-A82E | 408 | 0 | FEMALE | 60 | NA | G2 | T3-4 | N0 | Stage III |
| TCGA-EP-A12J | 570 | 0 | MALE | 62 | NA | G1 | T1 | NX | Stage I |
| TCGA-EP-A26S | 608 | 0 | MALE | 70 | 1 | G2 | T1 | N0 | Stage I |
| TCGA-EP-A2KA | 627 | 1 | FEMALE | 52 | 0 | G3 | T3-4 | NX | Stage III |
| TCGA-EP-A2KB | 596 | 1 | FEMALE | 46 | 1 | G2 | T1 | NX | Stage I |
| TCGA-EP-A3JL | 303 | 0 | MALE | 76 | 0 | G2 | T1 | NX | Stage I |
| TCGA-EP-A3RK | 363 | 0 | MALE | 73 | 0 | G2 | T3-4 | NX | Stage III |
| TCGA-ES-A2HS | 688 | 1 | MALE | 80 | 1 | G2 | T1 | NX | Stage I |
| TCGA-ES-A2HT | 438 | 1 | MALE | 54 | 1 | G2 | T1 | NX | Stage I |
| TCGA-FV-A23B | 1852 | 1 | FEMALE | 70 | NA |  | T2 | N0 | Stage II |
| TCGA-FV-A2QQ | 729 | 0 | MALE | 80 | NA | G2 | T1 | N0 | Stage I |
| TCGA-FV-A2QR | 581 | 1 | MALE | 75 | NA | G1 | T1 | N0 | Stage I |
| TCGA-FV-A3I1 | 247 | 1 | FEMALE | 81 | NA | G2 | T2 | N0 | Stage II |
| TCGA-FV-A3R2 | 194 | 1 | MALE | 75 | NA |  | T1 | NX | Stage I |
| TCGA-FV-A3R3 | 366 | 1 | FEMALE | 38 | 1 | G2 | T1 | NX | Stage I |
| TCGA-G3-A25T | 1553 | 0 | FEMALE | 45 | 0 | G2 | T3-4 | N0 | Stage III |
| TCGA-G3-A25U | 1636 | 0 | FEMALE | 63 | 0 | G3 | T1 | N0 | Stage I |
| TCGA-G3-A25V | 860 | 0 | MALE | 68 | 2 | G2 | T1 | N0 | Stage I |
| TCGA-G3-A25X | 1779 | 0 | MALE | 73 | 0 | G3 | T2 | N0 | Stage II |
| TCGA-G3-A25Y | 452 | 1 | FEMALE | 52 | 0 | G3 | T1 | N0 | Stage I |
| TCGA-G3-A25Z | 655 | 0 | MALE | 58 | 0 | G2 | T1 | N0 | Stage I |
| TCGA-G3-A3CH | 780 | 0 | MALE | 53 | 0 | G2 | T3-4 | N0 | Stage III |
| TCGA-G3-A3CI | 180 | 0 | MALE | 71 | 1 | G2 | T1 | N0 | Stage I |
| TCGA-G3-A3CJ | 594 | 0 | MALE | 52 | 0 | G2 | T2 | N0 | Stage II |
| TCGA-G3-A3CK | 585 | 0 | MALE | 61 | 0 | G2 | T1 | N0 | Stage I |
| TCGA-G3-A5SI | 768 | 1 | MALE | 44 | 1 | G2 | T2 | N0 | Stage II |
| TCGA-G3-A5SJ | 698 | 0 | MALE | 59 | 0 | G2 | T1 | NX | Stage I |
| TCGA-G3-A5SK | 744 | 0 | MALE | 58 | 0 | G1 | T1 | NX | Stage I |
| TCGA-G3-A5SL | 621 | 0 | MALE | 70 | 0 | G2 | T2 | NX | Stage II |
| TCGA-G3-A5SM | 520 | 0 | MALE | 58 | 1 | G3 | T2 | NX | Stage II |
| TCGA-G3-A6UC | 671 | 0 | MALE | 65 | 0 | G2 | T3-4 | N0 | Stage III |
| TCGA-G3-A7M5 | 447 | 0 | MALE | 76 | 0 | G2 | T1 | NX | Stage I |
| TCGA-G3-A7M6 | 632 | 0 | FEMALE | 60 | 0 | G3 | T1 | NX | Stage I |
| TCGA-G3-A7M7 | 361 | 0 | MALE | 65 | 0 | G1 | T1 | NX | Stage I |
| TCGA-G3-A7M8 | 430 | 0 | MALE | 31 | 0 | G1 | T1 | NX | Stage I |
| TCGA-G3-A7M9 | 56 | 1 | MALE | 70 | 0 | G2 | T3-4 | NX | Stage III |
| TCGA-GJ-A6C0 | 31 | 1 | FEMALE | 75 | NA | G2 | T2 | NX | Stage II |
| TCGA-HP-A5MZ | 91 | 1 | MALE | 78 | NA | G2 | T1 | NX | Stage I |
| TCGA-HP-A5N0 | 1147 | 1 | FEMALE | 90 | 0 | NA | NA | NX |  |
| TCGA-K7-A5RF | 631 | 0 | MALE | 64 | NA | G1 | T1 | NX | Stage I |
| TCGA-K7-A5RG | 519 | 0 | MALE | 66 | NA | G1 | T1 | NX | Stage I |
| TCGA-K7-A6G5 | 512 | 0 | MALE | 66 | 0 | G2 | T1 | N0 | Stage I |
| TCGA-KR-A7K0 | 65 | 1 | MALE | 65 | NA | G1 | T1 | N0 | Stage I |
| TCGA-KR-A7K7 | 951 | 0 | FEMALE | 61 | NA | G1 | T2 | N0 | Stage II |
| TCGA-KR-A7K8 | 906 | 0 | MALE | 57 | NA | G1 | T1 | N0 | Stage I |
| TCGA-LG-A6GG | 387 | 0 | FEMALE | 79 | 0 | G2 | T2 | NX | Stage II |
| TCGA-MI-A75C | 291 | 0 | MALE | 64 | 1 | G3 | T1 | N0 | Stage I |
| TCGA-MI-A75E | 507 | 0 | MALE | 61 | 1 | G2 | T3-4 | N0 | Stage III |
| TCGA-MI-A75G | 698 | 0 | MALE | 63 | 1 | G2 | T2 | N0 | Stage II |
| TCGA-MI-A75H | 747 | 0 | MALE | 77 | 1 | NA | NA | NX | NA |
| TCGA-MI-A75I | 630 | 0 | MALE | 61 | 1 | G1 | T2 | NX | NA |
| TCGA-MR-A520 | 229 | 0 | MALE | 58 | NA | G1 | T1 | NX | Stage I |
| TCGA-NI-A4U2 | 1791 | 1 | MALE | 71 | 0 | G1 | T3-4 | NX | Stage III |
| TCGA-PD-A5DF | 639 | 1 | FEMALE | 58 | NA | G2 | T3-4 | N0 | Stage III |
| TCGA-QA-A7B7 | 94 | 0 | MALE | 48 | 1 | G2 | T2 | NX | Stage II |
| TCGA-RC-A7S9 | 640 | 0 | FEMALE | 47 | 0 | G3 | T1 | N0 | Stage I |
| TCGA-RC-A7SB | 588 | 0 | MALE | 53 | 0 | G2 | T2 | N0 | Stage II |
| TCGA-RC-A7SF | 579 | 0 | MALE | 66 | 0 | G2 | T1 | N0 | Stage I |
| TCGA-RC-A7SK | 472 | 0 | MALE | 59 | 0 | G3 | T1 | N0 | Stage I |
| TCGA-RG-A7D4 | 1098 | 0 | MALE | 69 | NA | G2 | T2 | N0 | Stage II |
| TCGA-UB-A7MB | 601 | 0 | MALE | 24 | 0 | G3 | T2 | NX | Stage II |
| TCGA-UB-A7MC | 500 | 0 | MALE | 59 | 0 | G3 | T3-4 | N0 | Stage III |
| TCGA-UB-A7MD | 52 | 1 | MALE | 67 | 0 | G3 | T1 | N0 | Stage I |
| TCGA-UB-A7ME | 486 | 0 | MALE | 51 | 0 | G2 | T1 | NX | Stage I |
| TCGA-UB-A7MF | 214 | 1 | MALE | 56 | NA | G2 | T3-4 | NX | Stage III |
| OS: overall survival; ECOG PS: Eastern Cooperative Oncology Group Performance Status Score | | | | | | | | | |

**Supplementary Figure 1. GO-MF analysis for the coexpression mRNAs of CTD-2139B15.2 and CTC-297N7.9.** The cnetplots based on the coexpressed mRNAs of CTD-2139B15.2 and CTC-297N7.9 and the GO-MF terms enriched from the mRNAs were used to indicate the potential functions of CTD-2139B15.2 **(A)** and CTC-297N7.9 **(B)**. The brown node represents the enriched GO-MF term, with the size indicating the overall number of its included mRNAs. The other smaller nodes are the enriched mRNAs, and the node colors changing from green to red indicate the increased associations of the mRNAs with the specific lncRNA.
